# Hanbury-Brown and Twiss exchange and non-equilibrium-induced correlations in disordered, four-terminal graphene-ribbon conductor

**DOI:** 10.1038/s41598-018-32777-5

**Published:** 2018-10-08

**Authors:** Z. B. Tan, T. Elo, A. Puska, J. Sarkar, P. Lähteenmäki, F. Duerr, C. Gould, L. W. Molenkamp, K. E. Nagaev, P. J. Hakonen

**Affiliations:** 1 Low Temperature Laboratory, Department of Applied Physics, Aalto University, Espoo, Finland; 2 Physikalisches Institut (EP3), University of Würzburg, Würzburg, Germany; 3 Kotelnikov Institute of Radioengineering and Electronics, Russian Academy of Science, Moscow, Russia; 4 Institute of Solid State Physics, Russian Academy of Science, Chernogolovka, Russia

## Abstract

We have investigated current-current correlations in a cross-shaped conductor made of graphene. The mean free path of charge carriers is on the order of the ribbon width which leads to a hybrid conductor where there is diffusive transport in the device arms while the central connection region displays near ballistic transport. Our data on auto and cross correlations deviate from the predictions of Landauer-B*ü*ttiker theory, and agreement can be obtained only by taking into account contributions from non-thermal electron distributions at the inlets to the semiballistic center, in which the partition noise becomes strongly modified. The experimental results display distinct Hanbury – Brown and Twiss (HBT) exchange correlations, the strength of which is boosted by the non-equilibrium occupation-number fluctuations internal to this hybrid conductor. Our work demonstrates that variation in electron coherence along atomically-thin, two-dimensional conductors has significant implications on their noise and cross correlation properties.

## Introduction

Disordered graphene is an extraordinary tunable system for studying electrical conduction ranging from nearly ballistic transport^1,2^ to hopping conductivity^3–8^. In narrow graphene ribbon, in particular, the number of transport channels can be varied significantly by tuning charge density by gate voltage and conduction can be pinched off fully near the charge neutrality point (CNP). The elastic mean free path can be maintained relatively large compared with device dimensions, while the importance of localization and Coulomb interactions can be varied by adjusting the charge density^9–11^. Disorder in graphene can lead either to increase or decrease of shot noise, depending on the amount and nature of scatterers^12–15^. Thus, in graphene nanoribbon (GNR) systems, it is possible to study physics of current-current correlations in a regime where disorder can be tuned, which makes it an excellent platform for investigating noise properties of disordered conductors.

Shot noise originates from the granular nature of charge carriers, and it can be used as an independent test for the conduction mechanism^16,17^. However it is difficult to distinguish between different models of noise in graphene using two-terminal measurements because several of them give nearby strength, on the order of 0.3–0.4, when compared to Poissonian noise. One of the ways to overcome this difficulty is measuring the cross-correlated noise in multiterminal graphene systems.

In mesoscopic conductors with purely elastic scattering, there are two fundamental sources of noise^16^. The first source are fluctuations of the occupation numbers of electron states in the reservoirs. These fluctuations take place if the average occupation numbers are different from zero and 1 and they account for the equilibrium thermal noise at a finite temperature. This noise is proportional to the conductance of the system, and it is nonzero even for ballistic conductors, which lack any internal scattering. Another type of fluctuations is related to the scattering of particles inside the conductor, which partially reflects them back. These fluctuations are called *partition noise*, and it may be observed even at zero temperature if there is a net current through the conductor. This noise is typical of systems with tunneling or diffusive transport where the incoming electrons are described by a Fermi distribution. In our experiment, however, we have locally non-equilibrium distribution functions for electrons instead of Fermi distributions, which leads to clear modifications in the partitioning noise^18^. Hence, the goal of our paper is not to test the exactness of individual noise models but rather to test whether the structure under investigation satisfies the assumptions of a particular model.

Theoretical analysis of low frequency current-current cross correlations ${S}_{nm}=-\,\langle \delta {I}_{n}\,\delta {I}_{m}\rangle$ of current fluctuations $\delta {I}_{i}$ in terminals $i=n$ and $m$ in a diffusive cross geometry has been performed in refs^19^ and^20^ with virtually equivalent findings. In the semiclassical theory^20^, the spectral density of noise in a diffusive system is governed by the local distribution function. This function is sensitive to diffusion of electrons, which is dependent on the local conductance and geometry of the conductor. The semiclassical theory predicts similar behavior for shot noise (*i.e*. auto correlation ${S}_{nn}$) in all cross-shaped diffusive conductors with negligible resistance of the central region. The Fano factor, *i.e*. the ratio between the autocorrelation ${S}_{nn}$ and the Poissonian noise ${S}_{P}=e{I}_{n}$ related to current ${I}_{n}$ in terminal $n$, is found to remain at $F=1/3$, *i.e*. as for a single wire, when biasing is done at terminal $n$ and the other terminals are grounded. In particular, the semiclassical theory predicts additivity of cross correlations in such a cross-shaped conductor, which would mean the absence of Hanbury–Brown and Twiss (HBT) exchange effects^19^ in our sample.

In this paper, we report and analyze experimental results on auto and cross correlations in a graphene nanoribbon cross where the mean free path ${\ell }_{mfp}$ of charge carriers is on the order of the ribbon width. The relatively long ${\ell }_{mfp}$ makes this device as a hybrid conductor with diffusive transport in the device arms and ballistic propagation in the central connection region. Our data on current-current correlations deviate from the multiterminal noise predictions for diffusive systems^16,19,20^, and agreement can be obtained only by taking into account contributions from non-equilibrium charge carrier distributions that modify the occupation-number noise at the border of the central region connecting the arms of the cross. The presence of this additional noise contribution is corroborated by the observation of negative bend resistance which is a signature of ballistic propagation in the centre. Our experiments also reveal distinct HBT exchange correlations, the strength of which is boosted by the non-equilibrium occupation-number fluctuations internal to this hybrid conductor. The observed HBT effect varies substantially with gate voltage and it becomes very strong near the CNP.

**Figure 1:**
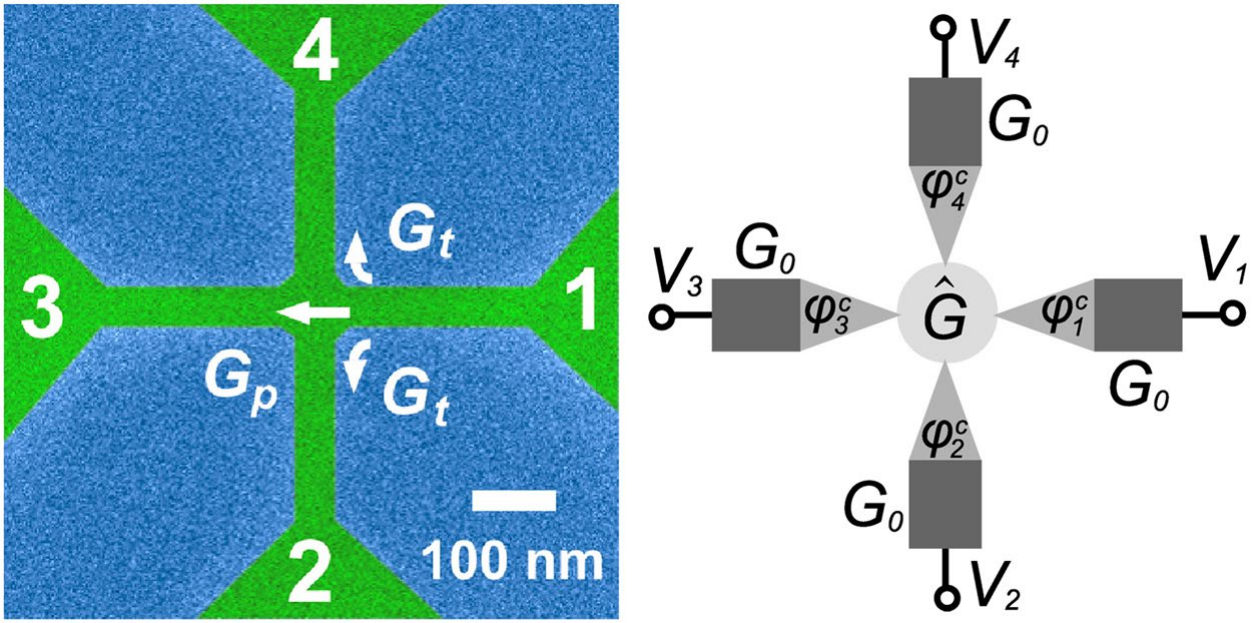
Left: False color scanning electron micrograph of the measured GNR sample; green color marks graphene and blue denotes the silicon oxide substrate. Terminals 1 and 3 were employed for cross correlation while bias was supplied via 2 and 4 in the HBT experiments. The white scale bar corresponds to 100 nm. The overlaid arrows define the straight and bent carrier paths with conductances of ${G}_{p}$ and ${G}_{t}$ in the central region, respectively, for electrons coming from terminal 1; the same definition of ${G}_{p}$ and ${G}_{t}$ repeats for electrons coming from each terminal. Right: Schematic illustration of our theoretical model with its most essential features: ${G}_{0}$ denotes the average arm conductance, $\widehat{G}$ describes the transport in the semiballistic central region, ${f}_{i}^{c}$ and ${\phi }_{i}^{c}$ mark the non-equilibrium distribution and the local voltage at the contact point between the diffusive arm and the central region, and ${f}_{0}(E)$ denotes the Fermi distribution. In the diffusive arm, the distribution function varies as ${f}_{i}(x,\,E)=(1-\frac{x}{L}){f}_{0}(E-e{V}_{i})+\frac{x}{L}\,{f}_{i}^{c}(E)$. For details, see text.

The basic assumption of diffusive transport theory is that the mean free path ${\ell }_{mfp}\ll \,{\rm{\min }}\,\{L,\,W\}$ compared with the length $L$ and width $W$ of the sample. The latter condition, however, is not well fulfilled in a narrow GNR, such as our sample illustrated in Fig. 1a. Deviations from finite size effects are estimated in ref.^19^, which predicts a small positive HBT exchange term on the order of $({\ell }_{mfp}/L)\,{G}_{0}\,eV$ for a metallic diffusive cross, where ${G}_{0}$ is the average arm conductance of the cross. This prediction turns out to have an opposite sign with respect to our experimental results, which are more in line with the behavior of a multiterminal chaotic quantum dot with internal ballistic transport^21^. Our measurements do reveal non-local conductance, which indicates that ${\ell }_{mfp}\simeq W$, and that the transport over the central area of the cross for many charge carriers is ballistic. Our results show that the noise properties of the system can be accounted for by the standard Langevin theory provided that the central region is considered as a distinct four-terminal ballistic conductor with nonequilibrium electron distributions ${f}_{i}^{c}$ at the internal terminals (see Fig. 1b), which also contributes to the noise through the occupation-number fluctuations of incident electrons^18^.

We investigated current-current cross correlations in a disordered cross-shaped graphene conductor. The length and the nominal width of the arms amount to $L\sim 240$ nm and $W\sim 50$ nm, respectively. A scanning electron micrograph of the actual measured sample is displayed in Fig. 1a. Figure 1b outlines the main features of the Langevin circuit model employed in our analysis. $\widehat{G}$ is a symmetric conductance matrix (see Eq. 5), which is composed of direct transmission with conductance ${G}_{p}$ through the central region and sideways transmission with conductance ${G}_{t}$ (left and right symmetric). The non-equilibrium distribution function ${f}_{i}^{c}$ at the contact point between the diffusive arm and the central region are calculated self-consistently using circuit analysis, and they govern the non-standard partition noise caused by the central region. In the diffusive arm, the distribution function varies as ${f}_{i}(x,\,E)=(1-\frac{x}{L}){f}_{0}(E-e{V}_{i})+\frac{x}{L}\,{f}_{i}^{c}(E)$ where $L$ is the length of the arm and ${V}_{i}$ is the applied voltage to the arm $i$.

## Theoretical Results

In contrast to ref.^19^ in which full quantum coherence was presumed over the system, we estimate that the coherence length is on the order of ${\ell }_{c}$ = 100–200 nm, which is based on weak localization experiments of ref.^22^ yielding ${\ell }_{c}\sim 200$ nm on similarly-fabricated micron-sized samples; our smaller estimate for ${\ell }_{c}$ is due to enhanced edge scattering in our 50-nm-wide GNRs, which leads to a decrease of the diffusion coefficient in the sample^23^. Consequently, ${\ell }_{c}$ is smaller than the length of the arms of the cross but larger than the size of its central region. This allows us to employ semiclassical circuit theory with Langevin noise generators for calculating incoherent noise contributions which originate from different parts of the graphene nanoribbon sample. Details of our Langevin model for a hybrid conductor (diffusive and ballistic transport in different parts) are presented in Methods section. In addition to regular ingredients of the diffusive Langevin formulation, our hybrid-conductor Langevin model (HCL model) contains a conductance matrix $\widehat{G}$ for the semiballistic central region (see Eq. 5), as well as separate potentials ${\phi }_{i}^{c}$ and distribution functions at the contact points of terminals $i$ to the central region. As we assume incoherent transport, the different noise contributions can be incoherently added. For the cross correlation ${S}_{13}$ biased by ${V}_{b}$ from terminal 1 with all the other terminals grounded we find

1 $${S}_{13}=-\,\langle \delta {I}_{1}\,\delta {I}_{3}\rangle =\frac{2{G}_{0}{G}_{p}^{2}(12{G}_{0}^{2}+21{G}_{0}{G}_{p}+32{G}_{p}^{2})}{\mathrm{3(}{G}_{0}+4{G}_{p}{)}^{4}}e{V}_{b},$$

where we have set ${G}_{t}={G}_{p}$ for simplicity as this corresponds to our experimental case. In the limit ${G}_{p}\to \infty$, we recover the diffusive limit. The calculated auto correlation with bias at terminal 1 and the other terminals grounded is given by

2 $${S}_{11}=2{G}_{0}{G}_{p}^{2}\frac{18{G}_{0}^{2}+45{G}_{0}{G}_{p}+32{G}_{p}^{2}}{{({G}_{0}+4{G}_{p})}^{4}}e{V}_{b}.$$

For the general formula for ${G}_{t}\ne {G}_{p}$, see the Methods section. The above ratio for ${S}_{13}/{S}_{11}$ depends on ${G}_{p}/{G}_{0}$ and, thus, it can be used to obtain information on ${G}_{p}/{G}_{0}$ of the graphene cross.

**Figure 2:**
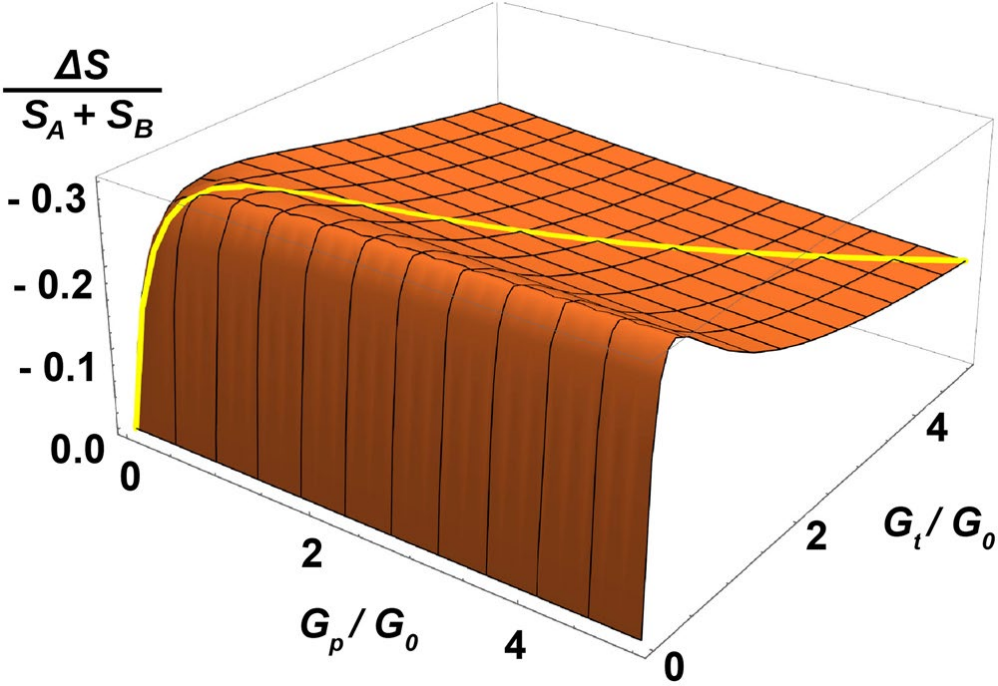
Theoretically calculated HBT effect ${\rm{\Delta }}S/({S}_{A}+{S}_{B})$ as a function of ${G}_{p}/{G}_{0}$ and ${G}_{t}/{G}_{0}$. In our analysis we are using the overlaid trace for ${\rm{\Delta }}S/({S}_{A}+{S}_{B})$ on the diagonal at which ${G}_{p}={G}_{t}$.

We define the Hanbury – Brown and Twiss exchange correlation term in accordance with ref.^19^ by ${\rm{\Delta }}S={S}_{C}-{S}_{A}-{S}_{B}$, where ${S}_{A}$, ${S}_{B}$, and ${S}_{C}$ denote the absolute values of the cross-correlated noise power spectra between terminals 1 and 3 in three different measurement configurations *A, B* and *C*: in the HBT configuration $A$, ($B$), bias was applied to terminal 2 (4) while the other terminals were connected to DC ground; in the case $C$, both 2 and 4 were biased and 1 and 3 DC-grounded. Using Eq. 1 and its variants for A, B, and C bias configurations, we obtain

3 $${\rm{\Delta }}S=-\,20\frac{{G}_{0}^{2}{G}_{p}^{3}}{{({G}_{0}+4{G}_{p})}^{4}}e{V}_{b}.$$

for the HBT exchange term. Our negative, non-zero result is in clear contrast with ${\rm{\Delta }}S=0$ obtained for regular diffusive systems by the semiclassical theory^20^, as well as ${\rm{\Delta }}S > 0$ predicted for ballistic graphene^24^. The calculated result for ${\rm{\Delta }}S/({S}_{A}+{S}_{B})$ is displayed in Fig. 2 on the plane spanned by ${G}_{p}$ and ${G}_{t}$. The regular diffusive behavior ${\rm{\Delta }}S=0$ is obtained in the limit ${G}_{p},\,{G}_{t}\to \infty$.

## Experimental Results

### Conductance

**Figure 3:**
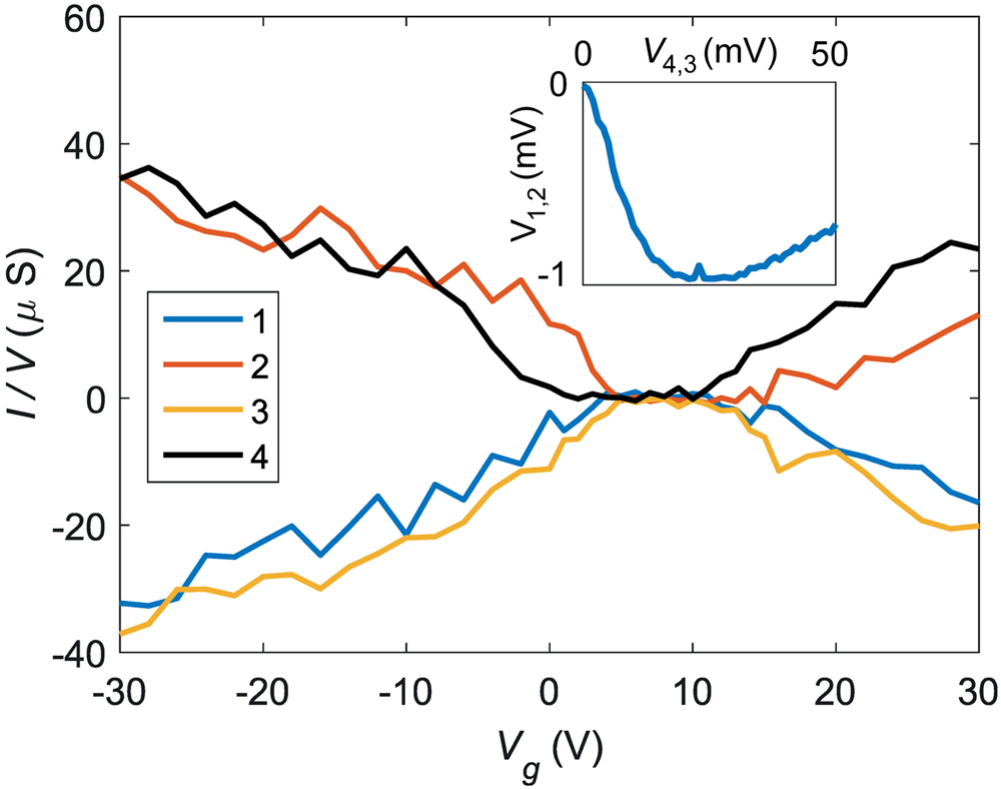
Conductance $G=I/V$ *vs*. ${V}_{g}$ measured at ${V}_{b}=30$ mV using the bias configuration C: Ingoing currents ${I}_{2}$ and ${I}_{4}$ are positive, while ${I}_{1} < 0$ and ${I}_{3} < 0$. The inset at ${V}_{g}=-\,30$ V displays negative bend voltage ${V}_{bend}={V}_{\mathrm{1,2}}$, where the bias is fed between terminals 4 and 3 and the voltage is measured across terminals 1 and 2.

We first characterized the sample conductances. The conductances of the arms were derived from the data for $I/V$ in Fig. 3 measured for the biasing configuration C, where the biasing leads 2 and 4 are seen with positive (ingoing) current, while currents in 1 and 3 are negative (outgoing). The currents in the four terminals are symmetric in general. Therefore, in semiclassical treatment, we may set the potential of the center of the cross to $V\mathrm{/2}$ in this measurement configuration. We obtain the arm conductances given in Table 1. The arm conductances in the proper diffusive regime far away from the Dirac point display symmetry within approximately ±6% % at ${V}_{g}=-\,30$ V and ±9% at ${V}_{g}=-\,10$ V. The symmetry of the four arms was also proven in measurements at ${V}_{g}=-\,30$ V in other configrations, in which ${g}_{\mathrm{1,2}}$, ${g}_{\mathrm{1,3}}$, ${g}_{\mathrm{1,4}}$, and ${g}_{\mathrm{2,4}}$ were determined with the remaining terminals floating, respectively. The difference of ${g}_{\mathrm{1,2}}$, ${g}_{\mathrm{1,3}}$, ${g}_{\mathrm{1,4}}$, and ${g}_{\mathrm{2,4}}$ was less than ±6%, which corroborates the symmetry of the four arms far away from the CNP. The measured conductivities correspond to a field effect mobility of ${\mu }_{FE}\simeq 500$ cm^2^/Vs, which is by a factor of two smaller than in the graphene cross experiments of ref.^25^. Some asymmetry in conductances is observed at ${V}_{g}\sim 0$ and ${V}_{g}\sim 15$ V. However, the asymmetry in these regions is bias dependent and its influence on cross correlations becomes reduced by using a fixed current-level correlation determination if necessary (For a linear cross-shaped conductor, when switching to measurement configuration C, the currents at terminals 1 and 3 double from the single source configurations A and B. For a nonlinear system, this is not the case unless one fixes the currents by adjusting voltages).

**Table 1:** Arm conductances (in *μ*S) at gate voltages ${V}_{g}=-\,10$ V and ${V}_{g}=-\,30$ V, indicating symmetry of the four arms.

*V*_*g*_	Arm 1	Arm 2	Arm 3	Arm 4
−10 V	22	20	22	24
−30 V	33	35	37	35

The conductances ${G}_{p}$ and ${G}_{t}$, defining the behaviour in the central region, were estimated from non-local measurements and the geometric dimensions. Though the arms of our sample are undoubtedly diffusive, the observed negative bend voltage is a clear sign of enhanced ballistic transport through the central area^26–28^. The observed bend voltage illustrated in Fig. 3 is rather small but it indicates nevertheless that part of the charge carriers traverse the central region ballistically. The bend voltage ${V}_{bend}$ can be calculated using the Landauer-Buttiker theory. The result using the parametrization of Fig. 1a reads

4 $${V}_{{\rm{bend}}}=\frac{{G}_{0}\,({G}_{t}-{G}_{p})}{{G}_{0}\,({G}_{p}+3\,{G}_{t})+8\,{G}_{t}\,({G}_{p}+{G}_{t})}\,{V}_{b}\mathrm{.}$$

The smallness of the measured bend voltage in Fig. 3 (approximately a few per cent of bias voltage) indicates that ${G}_{p}={G}_{t}$ within approximately ±20%. The size of the central region, taken as a square fitting within the middle of the cross, yields for the relative direct conductance ${G}_{p}/{G}_{0}=L/\sqrt{2}W=3.4$.

### Auto and Cross Correlations

Autocorrelation ${S}_{11}$ was investigated with bias ${V}_{b}$ in terminal 1 and the other terminals grounded. In this configuration, we found $F\simeq 0.4$, close to the values reported in ref.^25^ for a configuration with floating side terminals; similarly $F$ was increased near the CNP where the IV curves become strongly non-linear at small bias ${V}_{b} < 10$ mV. This Fano factor is higher than the universal value $F=1/3$ for diffusive systems.

**Figure 4:**
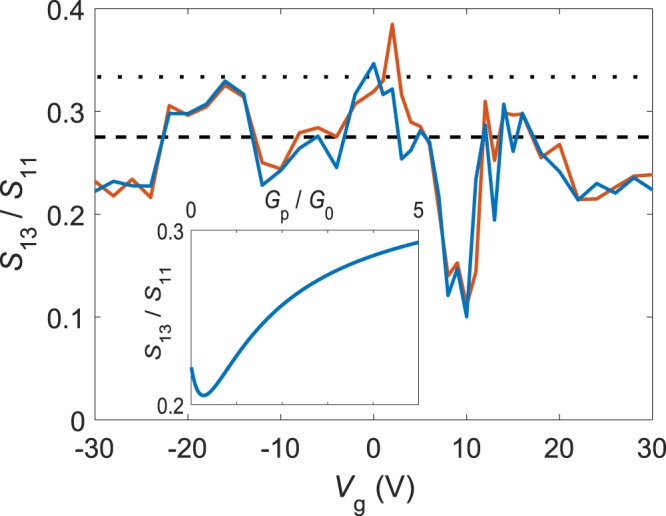
Ratio of ${S}_{13}/{S}_{11}$ vs. ${V}_{g}$ with bias applied via terminal 1 having the other terminals DC grounded. The two data sets, light and dark, relate to ${V}_{b}\lessgtr 0$, respectively: their difference is indicative of the small uncertainty in the data. The dashed line indicates the result from our HCL model with ${G}_{p}/{G}_{0}=3.4$. Our data deviates from the diffusive theory value 1/3 as shown in the dot line. The fluctuations in the data are related to universal noise fluctuations^30^. The inset displays the calculated behavior of ${S}_{13}/{S}_{11}$ *vs*. the ratio ${G}_{p}/{G}_{0}$ (at ${G}_{p}/{G}_{t}=1$).

For a symmetric diffusive cross with negligible resistance of the central region ${S}_{13}/{S}_{11}=1/3$. Our calculated result deviates from this universal diffusive-system value and our ratio depends on the significance of the occupation number noise induced by non-equilibrium distribution functions. The inset in Fig. 4 depicts the theoretical ratio as a function of ${G}_{p}/{G}_{0}$ at ${G}_{p}={G}_{t}$. When ${G}_{p}\to \infty$, our theory recovers the diffusive value ${S}_{13}/{S}_{11}=1/3$ as an extremum case, while in the limit of ${G}_{p}={G}_{t}\to 0$ we obtain 2/9. Hence, the non-equilibrium-induced occupation number noise will lead to a clear deviation from the diffusive behavior even though no asymmetry exists in the conduction and ${G}_{p}={G}_{t}$ at the crossing.

Figure 4 displays the measured ratio ${S}_{13}/{S}_{11}$ for our GNR cross. Off from the CNP, our measured ratio fluctuates between 0.22–0.32. We assign this variation to universal noise fluctuations^29^ which exist in all diffusive conductors^30^. These fluctuations allow only for a comparison of average values away from the CNP point. Our theoretical calculation for ${G}_{p}/{G}_{0}=3.4$ yields ${S}_{13}/{S}_{11}=0.275$, which agrees well with the average value of the experimental ratio at −$30{\rm{V}} < {{\rm{V}}}_{g} < -5{\rm{V}}$; the data at +$20{\rm{V}} < {{\rm{V}}}_{g} < +30{\rm{V}}$ would agree with a slightly smaller value for ${G}_{p}/{G}_{0}$, but the statistics here is too small to make definite conclusions. In the range of ${{\rm{V}}}_{g}=3\ldots +\,13$ V, in particular, the electrical transport is influenced by hopping conduction. Near the CNP ($8{\rm{V}} < {{\rm{V}}}_{g} < 10$ V), we find a decrease of ${S}_{13}/{S}_{11}$ down to 0.13–0.14 which is beyond the range of values produced by our HCL model. Inelastic hopping conduction via localized states near the CNP is a likely cause for the decrease of ${S}_{13}/{S}_{11}$.

### Hanbury – Brown and Twiss Exchange Correlations

**Figure 5:**
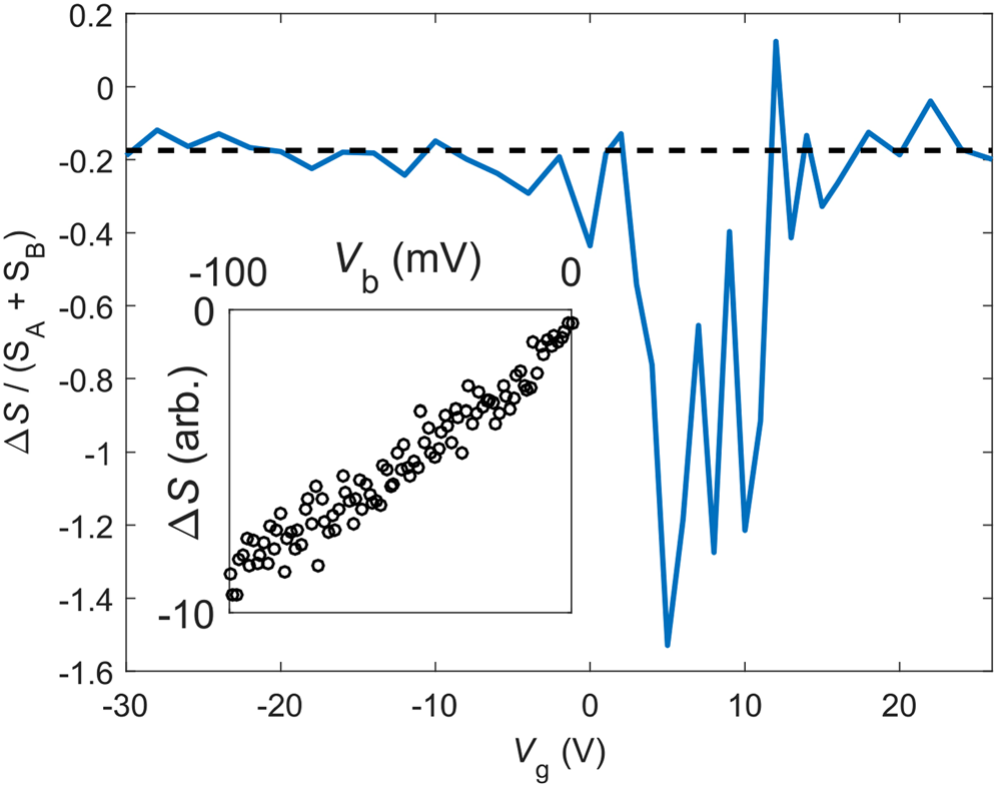
HBT exchange correction ${\rm{\Delta }}S$ vs. ${V}_{g}$ obtained from low-bias cross correlation experiments extrapolated to ${V}_{b}\to 0$. The solid line indicates our HCL model result ${\rm{\Delta }}S/({S}_{A}+{S}_{B})=-\,0.175$ using ${G}_{p}/{G}_{0}=3.4$. The inset displays the linear dependence of ${\rm{\Delta }}S$ on ${V}_{b}$ measured at ${V}_{g}=-\,30$ V.

Figure 5 displays our results for the HBT correlations. In order to compare the experimental results more accurately with theoretical predictions, we present the scaled HBT ratio ${\rm{\Delta }}S/({S}_{A}+{S}_{B})$ in Fig. 5. Our data display clearly a deviation from ${\rm{\Delta }}S/({S}_{A}+{S}_{B})=0$ which is the prediction of the regular diffusive theory. The dashed line in Fig. 5, obtained from Eq. (3) using ${G}_{p}/{G}_{0}=3.4$, corresponds to ${\rm{\Delta }}S=0.175\pm 0.007$ where the error estimate indicates the 20% uncertainty in the ratio ${G}_{p}/{G}_{t}$. The agreement between the model and the data is good in the regime where the charge density in the sample is large. However, there is a strong modification of the HBT exchange factor near the Dirac point. The strength of this change, however, cannot be captured by our HCL model. In our theoretical model (see Fig. 2), ${\rm{\Delta }}S$ is seen to vary with the ratio of ${G}_{p}/{G}_{0}$ which is likely to be modified near the CNP point. Thereby, a moderate decrease in ${\rm{\Delta }}S$ could be understood in terms of a stronger gate dependence in the conductance in the central region compared to that in the arms. Such a mechanism, however, could only account for results with absolute values $|{\rm{\Delta }}S|/({S}_{A}+{S}_{B}) < 0.30$, which clearly falls short from our measured results.

## Discussion

Our results on the auto and cross correlation power show linear bias dependence at currents <1 $\mu$A (see the inset of Fig. 5), which becomes slightly weaker at currents well above 1 *μ*A where inelastic scattering starts to take place. When inelastic processes are important (inelastic length ${l}_{in}\lesssim L$), shot noise in graphene is reduced by the most strongly coupled energy relaxation processes, i.e. either by impurity-assisted acoustic phonon collisions or by optical phonons^31–34^. Inelastic processes were strongest in our work near the CNP, as rather large voltages were needed for biasing. However, the final quoted results were always obtained in the limit ${V}_{b}\to 0$, which eliminates any effects due to inelastic phonons.

The shot noise results away from the CNP with $F\simeq 0.4$ are in agreement with the theoretical results for disordered graphene ribbons^13^. In ref.^25^ it was concluded that these results are in accordance with Gaussian disorder having a dimensionless strength of ${K}_{0}\approx 10$, which meant that the conductance is strongly affected by disorder^13^. However, almost the same result is obtained in our HCL model with diffusive arms and ballistic central region. Indeed, the calculations for ${G}_{p}={G}_{t}=3.4\,{G}_{0}$ give ${F}_{f}=0.367$ for the floating side terminals and ${F}_{g}=0.394$ for the three grounded ones (See Methods). This excellent agreement between our HCL Langevin circuit theory and the experimental results indicates that transport in graphene is well amenable to analysis using semiclassical methods having only a few overall parameters.

Besides shot noise, fine agreement is found between the measured HBT exchange and our HCL model at large charge density. This indicates that our theory is able to capture well the correlations that are generated by two particle scattering^16^ in a disordered graphene conductor. Our results signify that not only the geometry is important for HBT correlations but also there is a need to know the local distribution functions driving partitioning in multiterminal graphene conductors. The HBT correlations become more complex when approaching the CNP with localized states. The strong growth of the absolute value of HBT exchange effect near the CNP is presumably caused by Coulomb blockade and tunneling conductance becoming more important. For a metallic island connected to four metallic leads by four tunnel junctions, we have measured ${\rm{\Delta }}S/({S}_{A}+{S}_{B})=-\,1$ within 3%^35^. In addition, we did analyze whether enhanced electron-electron interactions near the CNP could account for the increased HBT effect at small charge density. However, a calculation using local hot electron distribution functions (without a ballistic center) yields ${\rm{\Delta }}S/({S}_{A}+{S}_{B})=-\,0.295$, which is clearly different from the measured results, both near the CNP and far away from it.

To conclude, we have studied cross correlations in a diffusive, disordered graphene conductor where the elastic mean free path is on the order of the feature size of the geometric layout. Even though only weak non-local transport features can be observed due to ballistic propagation in the central region of the graphene cross, their presence promotes non-equilibrium-induced occupation-number noise that has an essential influence on the current-current correlations in such a hybrid multiterminal conductor. As a consequence, the noise properties of this disordered system cannot be treated by standard diffusive theories. By inclusion of a combinational occupation noise, i.e., partitioning noise driven by current-generated non-equilibrium distribution functions, remarkable agreement is obtained between our semiclassical (HCL) model and the measured noise properties, including the Hanbury–Brown and Twiss exchange effects in the transport regime where charge density is large. Altogether, our experiment casts important light on transport phenomena in multiterminal graphene conductors where complementary quantum noise issues due to local ballistic propagation have to be taken into account to treat these hybrid conductors with simultaneous diffusive and ballistic characteristics. The conclusions of our work are relevant also to other atomically-thin, two-dimensional conductors with similar characteristics^36^.

## Methods

### Experimental methods

The ribbon samples were fabricated from micromechanically cleaved graphene on a heavily *p*-doped substrate with a 280-nm thick layer of SiO_2_. Metallic leads to contact the graphene sheet were first patterned using standard e-beam lithography followed by a Ti(2 nm)/Au(35 nm) bilayer deposition. After lift-off in acetone, a second lithography step facilitated patterning of the GNRs. Our measurements down to 50 mK were performed on a dry dilution refrigerator. Standard lock-in techniques using DL Instruments 1211 preamplifier followed by a Stanford SR830 lock-in amplifier were employed for conductance measurements. The IV measurements have been done by using Agilent 33120a generator and 34401a multimeter.

Our cross and auto correlation measurement system operates over frequencies *f*_*BW*_ = 600–900 MHz^37^. This frequency is typically well above any fluctuator noise due to switching in transmission eigenvalues at the contacts^31^. Still, the employed frequency range is low enough to correspond to zero-frequency noise because the frequency is low compared with the internal $\mathrm{1/}RC$ scale and the temperature. An aluminum tunnel junction was used for calibration of the noise spectrometers^38^. Auto and cross correlations were measured using two software defined digital radio receivers^37^. For IV curves with significant non-linearity, ${S}_{nm}$ values were first derived as a function of current in the limit $I\to 0$. The resulting reading of $d{S}_{nm}/dI$ was converted to $d{S}_{nm}/d{V}_{b}$ by using the measured differential conductance $dI/d{V}_{b}$. Note that we always take the opposite of the cross correlations when $n\ne m$, which makes ${S}_{nm}$ positive as all these non-diagonal correlations are negative in a fermionic system.

### Theoretical modeling

The system we address is of hybrid type as its different parts exhibit both diffusive and ballistic conduction. It represents a conducting cross whose arms are much longer than the elastic mean free path ${\ell }_{mfp}$, but the central region at the intersection is on the order of ${\ell }_{mfp}$. We also assume that the motion of electrons along the arms is incoherent. Therefore it is possible to adopt a circuit model of the system shown in Fig. 1b. The equivalent circuit of the system consists of four diffusive wires shown by dark rectangles, the central ballistic region shown by the white circle, and four transition regions between the diffusive wires and the ballistic region, which serve as “reservoirs” with nonequilibrium electron distributions acting on the central region. The length of these “reservoirs” is assumed to be on the order of ${\ell }_{mfp}$ and hence their resistance is negligible when compared with $\mathrm{1/}{G}_{0}$ of the diffusive arms. To calculate the average current in each arm, we treat the four diffusive arms and the central connecting region as separate elements of the circuit, and the average current in each arm can be found from a system of Kirchhoff’s circuit laws. The four arms of the cross are modelled as two-terminal resistors with equal conductances ${G}_{0}$, The ballistic central region may be treated as a four-terminal conductor with $2m+n$ reflectionless channels originating from each arm. Of these channels, $n$ go straight ahead into the opposite arm, while $m$ channels turn left and right, respectively. Hence the conductance matrix of the central region may be written in the form

5 $$\widehat{G}=(\begin{array}{cccc}2\,{G}_{t}+{G}_{p} & -{G}_{t} & -{G}_{p} & -{G}_{t}\\ -{G}_{t} & 2\,{G}_{t}+{G}_{p} & -{G}_{t} & -{G}_{p}\\ -{G}_{p} & -{G}_{t} & 2\,{G}_{t}+{G}_{p} & -{G}_{t}\\ -{G}_{t} & -{G}_{p} & -{G}_{t} & 2\,{G}_{t}+{G}_{p}\end{array})$$

where ${G}_{p}=2m{e}^{2}/h$ and ${G}_{t}=2n{e}^{2}/h$. If the electrical potential in arm $i$ at the crossing is ${\phi }_{i}^{c}$, the total current flowing from this arm into the other arms equals

6 $${I}_{i}=\sum _{j}\,{\widehat{G}}_{ij}\,{\phi }_{j}^{c}.$$

On the other hand, this current is given by the Ohm’s law in the diffusive arm $i$

7 $${I}_{i}={G}_{0}\,({V}_{i}-{\phi }_{i}^{c}),$$

where ${V}_{i}$ is the external voltage applied to the outer end of the arm. Equations (6) and (7) form a full system for finding the currents ${I}_{i}$ in each arm of the cross.

The two-terminal resistance between the opposite ends of the cross is calculated by setting ${V}_{1}={V}_{b}$, ${V}_{3}=0$, and ${I}_{2}={I}_{4}=0$. Solving Eqs (6) and (7) for ${I}_{1}$ readily gives

8 $${R}_{2t}\equiv {V}_{b}/{I}_{1}=\frac{{G}_{0}+2\,({G}_{p}+{G}_{t})}{{G}_{0}\,({G}_{p}+{G}_{t})}.$$

To calculate the bend voltage, it is sufficient to substitute ${V}_{3}=0$, ${V}_{4}={V}_{b}$, and ${I}_{1}={I}_{2}=0$ into the system (6)–(7) and solve it for ${V}_{1}$ and ${V}_{2}$. As a result, one obtains

9 $${V}_{bend}={V}_{1}-{V}_{2}=\frac{{G}_{0}\,({G}_{t}-{G}_{p})}{{G}_{0}\,({G}_{p}+3{G}_{t})+8{G}_{t}\,({G}_{p}+{G}_{t})}\,{V}_{b}.$$

The current flowing through arms 4 and 3 is

10 $${I}_{4}=-\,{I}_{3}=\frac{4{G}_{0}\,{G}_{t}\,({G}_{p}+{G}_{t})}{{G}_{0}\,({G}_{p}+3{G}_{t})+8{G}_{t}\,({G}_{p}+{G}_{t})}{V}_{b},$$

and the bend resistance equals

11 $${R}_{b}\equiv ({V}_{2}-{V}_{1})/{I}_{4}=\frac{1}{4}\,\frac{{G}_{t}-{G}_{p}}{{G}_{t}\,({G}_{p}+{G}_{t})}.$$

If the motion of electrons in the diffusive arms is incoherent, the arms of the cross and the central region may be considered as independent sources of noise. The fluctuations of current in the arms are conveniently described by the semiclassical Langevin equation

12 $$\delta {I}_{i}=\delta {I}_{i}^{ext}({x}_{i})+L{G}_{0}\frac{d\delta {\phi }_{i}({x}_{i})}{d{x}_{i}},$$

where $\delta {I}_{i}^{ext}({x}_{i})$ is the extraneous Langevin current, ${x}_{i}$ is the coordinate along the arm, $L$ is its length, and $\delta {\phi }_{i}({x}_{i})$ is the fluctuation of electric potential in this arm. The correlation function of the Langevin currents is^39^

13 $$\langle \delta {I}_{i}^{ext}(x)\,\delta {I}_{j}^{ext}(x^{\prime} )\rangle =4\,{\delta }_{ij}\,\delta (x-x^{\prime} )\,L{G}_{0}\,\int \,d\varepsilon \,{f}_{i}(x,E)\,[1-{f}_{i}(x,E)],$$

where ${f}_{i}(x,\,E)$ is the distribution function of electrons in arm $i$. An integration of Eq. (12) over $x$ with the condition $\delta {\phi }_{i}(0)=0$ brings it to the form

14 $$\delta {I}_{1}={G}_{0}\,\delta {\phi }_{i}^{c}+{\int }_{0}^{L}\,\frac{dx}{L}\,\delta {I}_{i}^{ext}(x),$$

where $\delta {\phi }_{i}^{c}\equiv \delta {\phi }_{i}(L)$ is the potential fluctuation of the reservoir in arm $i$ at the crossing.

On the other hand, the central ballistic region is also a source of noise. Although there is no electron backscattering and thus no true partition noise there, it can generate the noise due to occupation-number fluctuations in its “reservoirs” because the distribution functions ${f}_{i}^{c}(E)\equiv {f}_{i}(L,\,E)$ at the ends of the corresponding arms are nonequilibrium and different from zero and 1 in a range of energies. The fluctuation of current flowing from arm $i$ into the rest of arms equals

15 $$\delta {I}_{i}=\sum _{j}\,{G}_{ij}\,\delta {\phi }_{j}^{c}+\delta {\tilde{I}}_{i}^{ext},$$

where $\delta {\tilde{I}}_{i}^{ext}$ are extraneous random currents generated at the crossing due to the nonequilibrium distribution of incident electrons with the correlation function^16^

16 $$\langle \delta {\tilde{I}}_{i}^{ext}\,\delta {\tilde{I}}_{j}^{ext}\rangle =2\,{G}_{ij}\,\int \,dE\,[{f}_{i}^{c}\,(1-{f}_{i}^{c})+{f}_{j}^{c}\,(1-{f}_{j}^{c})].$$

The values of ${f}_{i}^{c}$ may be obtained from a system of equations similar to Eqs (6) and (7) with ${f}_{i}^{c}$ in place of ${\phi }_{i}^{c}$ and ${f}_{0}(E-e{V}_{i})$ in place of ${V}_{i}$ where ${f}_{0}(E)$ is the Fermi distribution function. The distribution function of electrons in the arms is governed by simple diffusion at a given energy, which yields a linear combination of distributions at its ends

17 $${f}_{i}(x,E)=(1-\frac{x}{L}){f}_{0}(E-e{V}_{i})+\frac{x}{L}\,{f}_{i}^{c}(E).$$

The system of equations (14) and (15) has to be solved for $\delta {I}_{i}$ and $\delta {I}_{j}$, and then the correlation function ${S}_{ij}=-\,\langle \delta {I}_{i}\,\delta {I}_{j}\rangle$ has to be calculated using Eqs (13) and (16).

First of all, we calculate the two-terminal Fano factor for the case where the current flows only through arms 1 and 3, whereas side arms 2 and 4 are floating. Hence one has to set ${V}_{1}={V}_{b}$, ${V}_{3}=0$ and ${I}_{2}={I}_{4}=0$ for the averages and $\delta {V}_{1}=\delta {V}_{3}=\delta {I}_{2}=\delta {I}_{4}=0$ for the fluctuations. This gives us the Fano factor in the form

18 $${F}_{f}\equiv {S}_{11}/|e{I}_{1}|=\frac{2}{3}\,({G}_{p}+{G}_{t})\,\frac{6\,{G}_{0}^{2}+9\,{G}_{0}\,({G}_{p}+{G}_{t})+4\,{({G}_{p}+{G}_{t})}^{2}}{{[{G}_{0}+2({G}_{p}+{G}_{t})]}^{3}}.$$

It is easily seen that ${F}_{f}$ depends only on the ratio between ${G}_{p}+{G}_{t}$ and ${G}_{0}$. It tends to zero as for a purely ballistic system when this ratio is small and approaches the 1/3 value for a diffusive conductor when ${G}_{p}+{G}_{t}\gg {G}_{0}$. The value passes through a maximum ${F}_{f}\approx 0.48$ at $({G}_{p}+{G}_{t})/{G}_{0}=(\sqrt{5}-1)/2\approx 0.62$, while it equals 0.367 for ${G}_{p}={G}_{t}=3.4\,{G}_{0}$. The latter value is larger than the shot noise for diffusive conductor with purely elastic scattering, but somewhat smaller than the noise in the hot-electron regime.

In a configuration where the voltage is applied to terminal 1 and the rest of terminals are grounded, one sets $\delta {V}_{i}=0$ for all $i$. The general expression for the Fano factor is too cumbersome, and we present it here only for the particular case of ${G}_{p}={G}_{t}$, where it reads

19 $${F}_{g}\equiv {S}_{11}/|e{I}_{1}|=\frac{2}{3}\,{G}_{p}\frac{18\,{G}_{0}^{2}+45\,{G}_{0}{G}_{p}+32\,{G}_{p}^{2}}{{({G}_{0}+4{G}_{p})}^{3}}.$$

The ${F}_{g}({G}_{p}/{G}_{0})$ curve is similar in shape to ${F}_{f}({G}_{p}/{G}_{0})$ but lies higher and reaches its maximum ${F}_{g}=0.65$ at ${G}_{p}/{G}_{0}\approx 0.24$. For our particular values ${G}_{p}={G}_{t}=3.4\,{G}_{0}$, ${F}_{g}$ equals 0.394 as mentioned in the main text.

In a similar way, one calculates ${S}_{13}^{A}$ for ${V}_{1}={V}_{3}={V}_{4}=0$ and ${V}_{2}={V}_{b}$, ${S}_{13}^{B}$ for ${V}_{1}={V}_{2}={V}_{3}=0$ and ${V}_{4}={V}_{b}$, and ${S}_{13}^{C}$ for ${V}_{1}={V}_{3}=0$ and ${V}_{2}={V}_{4}={V}_{b}$. The resulting exchange term in the noise equals

20 $${\rm{\Delta }}S\equiv {S}_{13}^{C}-{S}_{13}^{A}-{S}_{13}^{B}=-\tfrac{20}{3}\tfrac{{G}_{0}^{2}\,{G}_{t}^{2}\,(10\,{G}_{t}+2\,{G}_{p}+3\,{G}_{0})({G}_{0}\,{G}_{p}+2\,{G}_{p}\,{G}_{t}+2\,{G}_{t}^{2})}{{({G}_{0}+2{G}_{p}+2{G}_{t})}^{2}{({G}_{0}+4{G}_{t})}^{4}}\,e{V}_{b}.$$

This formula yields Eq. 3 in the main text using ${G}_{t}={G}_{p}$.

## Data Availability

The datasets generated and analysed during the current study are available from the corresponding author on reasonable request.
